# Populations of the coral species *Montastraea cavernosa* on the Belize Barrier Reef lack vertical connectivity

**DOI:** 10.1038/s41598-019-43479-x

**Published:** 2019-05-10

**Authors:** Ryan J. Eckert, Michael S. Studivan, Joshua D. Voss

**Affiliations:** Florida Atlantic University, Harbor Branch Oceanographic Institute, 5600 N US Highway 1, Fort Pierce, FL 34946 USA

**Keywords:** Ecological genetics, Molecular ecology, Population genetics

## Abstract

Larval connectivity among and within coral reefs is important for sustaining coral metapopulations, enhancing ecosystem resilience through species and genetic diversity, and maintaining reef ecosystems’ structure and functions. This study characterized genetic structure and assessed horizontal and vertical connectivity among populations of the ubiquitous gonochoric broadcast spawning coral *Montastraea cavernosa* in Belize. Using nine polymorphic microsatellite loci, we genotyped *M*. *cavernosa* colonies from four depth zones at four study sites within Belizean marine management zones. Study sites were selected within South Water Caye Marine Reserve (3 sites) and Glover’s Reef Marine Reserve (1 site). Strong contemporary genetic differentiation was observed between relatively shallow *M*. *cavernosa* populations (10 m, 16 m) and relatively deep (25 m, 35 m) populations, coinciding with a transition from reef crest to reef slope. These results were consistent across both marine reserves. Vertical and horizontal migration models suggest that all populations were historically panmictic, with little unidirectional migration. The relative local isolation of shallow and mesophotic *M*. *cavernosa* populations in Belize, coupled with the importance of Belize’s upper mesophotic populations as potential larval sources for other areas in the Tropical Western Atlantic, reinforces the need for management strategies that conserve coral populations across all depth zones.

## Introduction

Given the multitude of anthropogenic threats to coral reefs and steady declines of shallow coral cover observed over the past several decades^1–5^, deeper reefs have been proposed as potential refuges for at-risk, shallow reefs^1,6,7^. Glynn^1^ first introduced a hypothesis positing that deep reefs may function as refuge habitats for shallow reefs, which has been further developed and termed the Deep Reef Refugia Hypothesis^6,8–10^. This hypothesis relies heavily on the assumption that deeper coral reefs are able to provide viable offspring to recolonize degraded shallow coral reefs^8^. In particular, depth-generalist coral species found along depth gradients encompassing both shallow and mesophotic coral ecosystems (MCEs; ~30–150 m) are potential candidates for contributing larvae to shallow coral reef areas. In the Tropical Western Atlantic (TWA), it is estimated that at least 25–40% of coral species found on shallow coral reefs also occur on MCEs^8,9^. While quantification of larval dispersal, recruitment, and survivorship *in situ* is a challenging feat, assessing similarities in genomic DNA among shallow and mesophotic coral populations can provide evidence of contemporary and historical patterns of connectivity, and evaluate the potential for mesophotic coral populations to replenish declining communities of degraded shallow coral reefs.

Polymorphic molecular markers, including microsatellites, can be used to infer genetic differences among coral populations and to estimate genetic structure within populations. Studies in the TWA have largely examined horizontal connectivity through the estimation of gene flow among sites across broad spatial scales^11–14^. Multiple studies have also implemented genetic methodologies to quantify vertical connectivity of depth-generalist scleractinian coral species in order to address the refuge potential of MCEs^9,15–18^. Studies focusing on the scleractinian coral species *Montastraea cavernosa* (Linnaeus, 1767) have demonstrated varying degrees of connectivity between MCEs and shallow coral reefs. *M*. *cavernosa* is found throughout the TWA, including most reefs off the coast of Belize. An extreme depth-generalist, *M*. *cavernosa* is found across a variety of habitats ranging from 1–113 m in depth^8,19^. In a broad-scale study of *M*. *cavernosa* populations in the TWA, populations in the U.S. Virgin Islands and Bermuda showed no differentiation across depth zones, while populations in South Florida demonstrated evidence of genetic structure across depth zones^16^. Similarly, while *M*. *cavernosa* populations in both Little Cayman Island and San Salvador, Bahamas were differentiated by depth, populations in Lee Stocking Island, Bahamas were relatively well-connected^15^. *M*. *cavernosa* populations in the northwestern Gulf of Mexico (NW GOM) are likely a single panmictic population, with no genetic structure between shallow and mesophotic depth zones, while in Belize and southwest Florida, populations were genetically differentiated by depth^18^. While these studies have focused on large geographic scales (100 s of km), there has been little work focused on small scale, local population structure and connectivity of scleractinian species in Belize.

**Figure 1:**
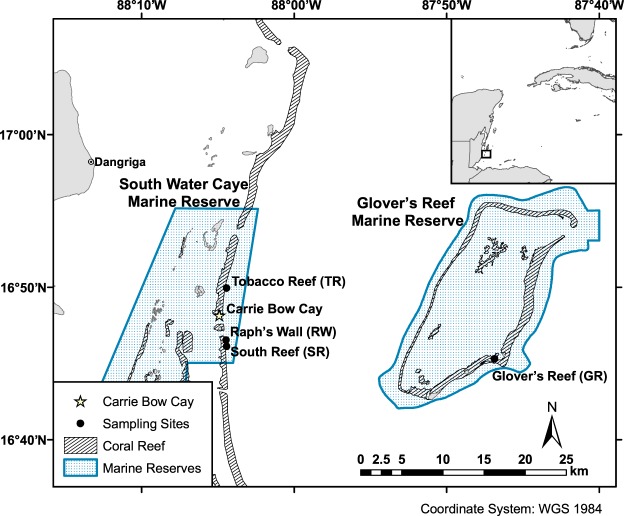
Map of Belize Barrier Reef with sampling sites within South Water Caye and Glover’s Reef Marine Reserves overlaid. Coral reef habitat and MPA shapefiles adapted from Meerman and Clabaugh^72^.

**Figure 2:**
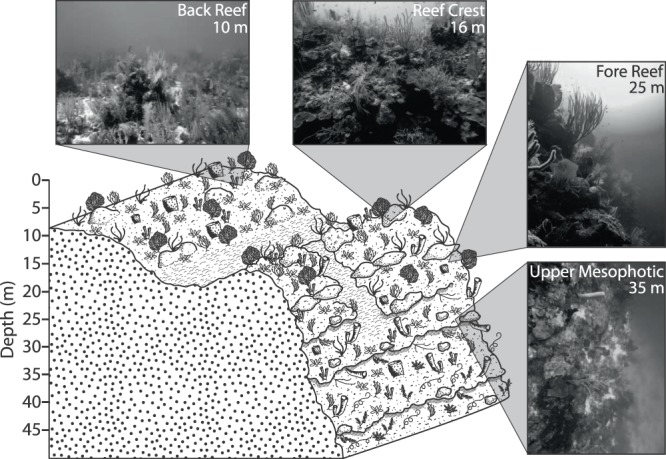
Diagram of a typical sampling site showing generalized reef geomorphology along sampling depths. Inset photographs are representative of typical habitat at sampling depths.

The barrier reef off the coastline of Belize (i.e. the Belize Barrier Reef) connects the reefs of Guatemala and Honduras to the south and east with the reefs of Mexico to the north, collectively forming the greater Mesoamerican Reef. The shoreward side of the reef is separated from the coast by a lagoon which ranges from 20–40 km in width and 1–65 m in depth, containing hundreds of patch reefs^20,21^. Reef margins on the barrier reef surrounding Carrie Bow Cay and the seaward side of Glover’s Reef Atoll (Fig. 1) typically exhibit spur and groove structures to a depth of 20–33 m, with a near vertical step from 30–37 m, and a slope to a reef wall extending to depths beyond 100 m^20^ (Fig. 2). A network of seventeen multi-use Marine Protected Areas (MPAs) has been established in Belize since 1982. These MPAs span over 3,600 km^2^ (~10%) of the country’s territorial seas, ~530 km^2^ of which are designated no-take zones^22^. The barrier reef surrounding Carrie Bow Cay and all of Glover’s Reef Atoll lie within South Water Caye and Glover’s Reef Marine Reserves, respectively. Both reserves have multiple levels of management ranging from general use to highly restrictive preservation or wilderness zones. Sites within these marine reserves benefit from restrictions to access, fishing, and anchoring^23,24^. Despite the large body of literature available on the Belize Barrier Reef, there have been relatively few studies of scleractinian corals within the depth range of MCEs^18,25^, and even fewer focused on population genetics within this depth range. The majority of information available on scleractinian distribution below 30 m was published in the 1970s from submersible dives by James and Ginsberg^20^ and in the 1980s from open circuit SCUBA dives near Carrie Bow Cay^26,27^. Furthermore, only one study^18^ of the several recent studies examining scleractinian population genetics and connectivity^13,14^ included samples from Belizean MCEs.

This study investigated the patterns of genetic connectivity among populations of *M*. *cavernosa* across a small spatial scale, specifically targeting reefs within the nearby South Water Caye and Glover’s Reef Atoll Marine Reserves. A multi-regional analysis of *M*. *cavernosa* connectivity reported little connectivity between mesophotic (~35 m) and relatively shallow (~15 m) sites in Belize, despite short lateral separation between populations (~10 m of horizontal distance)^18^. Through the sampling of an additional site and depth zones we sought to describe small scale vertical and horizontal connectivity across a depth gradient within existing MPAs to evaluate if populations coincided with depth-based habitat characterization (i.e. “shallow” and “mesophotic”) as potentially indicated in previous work from the region^18^.

## Results

### Genetic differentiation and population structure along a depth gradient

**Figure 3:**
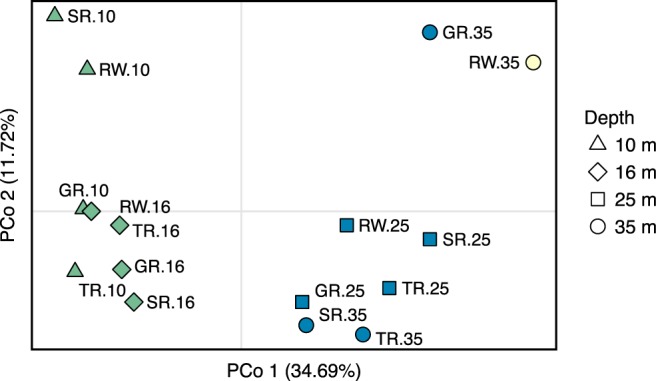
Principal coordinates analysis (PCoA) based on Nei genetic distance (*D*_A_) matrix of pairwise population comparisons. Shape denotes depth zone and color denotes the dominant genetic cluster (*K* = 3) within each population, as determined by structure analysis. Populations are labeled next to points, formatted as SITE.DEPTH (site abbreviations listed in Table 2).

**Figure 4:**
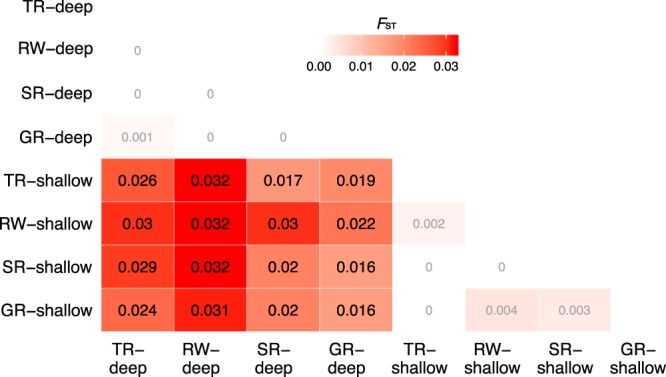
Heat map showing pairwise population differentiation through estimation of fixation index (*F*_ST_) between combined “shallow” (10 m and 16 m) and “deep” (25 m and 35 m) populations. Values within cells are estimated *F*_ST_; intensity of red coloration corresponds with increasing *F*_ST_; bolded values denote significant differentiation between populations (after FDR correction; α = 0.05). Site abbreviations listed in Table 2.

Analysis of molecular variance showed low but significant differentiation across sampled *M*. *cavernosa* populations (AMOVA; 1.60%, *df*_15,239_, SS = 78.81, *p* = 0.0001), with the highest genotypic variation being among individuals within populations (12.26%, *df*_223,239_, SS = 817.80). Principal coordinates analysis (PCoA) revealed similarities and clustering of the 10 m and 16 m depth zones and 25 m and 35 m depth zones, respectively (Fig. 3). Pairwise population *F*_ST_ comparison supported the PCoA patterns, revealing that 10 m and 16 m populations were similar to one another but significantly differentiated from 25 m and 35 m populations (Supplementary Fig. S1). When samples were combined into “shallow” (10 m and 16 m) and “deep” (25 m and 35 m) populations, every deep-shallow pairwise comparison demonstrated significant differentiation (Figs. 4 and S1). Additionally, *F*_ST_ comparisons between populations at Raph’s Wall and all other populations were among the most highly differentiated pairwise comparisons (Fig. 4). A Mantel test suggested no significant correlation between geographic and genetic distances in the sampling region (*R*^2^ = 0.0069, *p* = 0.21); geographic distances between sampling sites ranged from 0.796–32.322 km.

**Figure 5:**
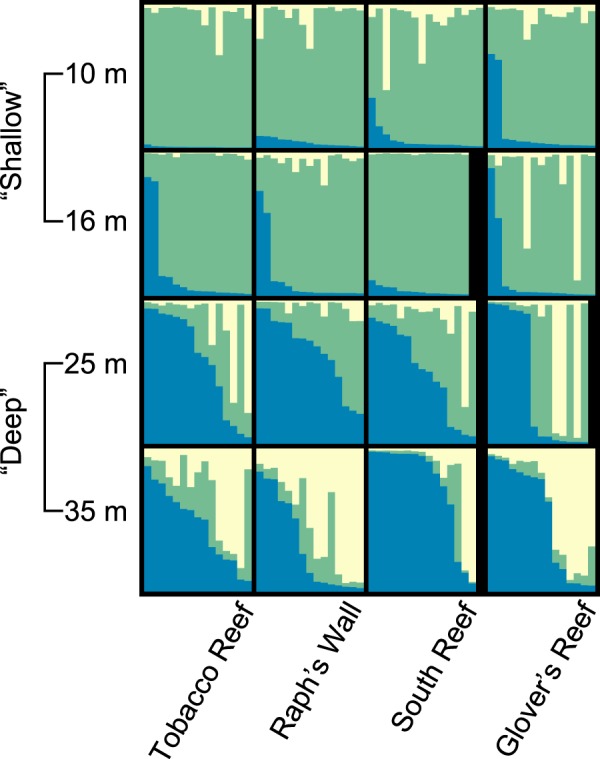
Genetic structure plots for *Montastraea cavernosa* populations from 4 depth zones across 4 reef sites in Belize. Each vertical bar is an individual *M*. *cavernosa* sample showing percent membership to each of 3 proposed genetic clusters (*K*) represented as Blue, Green, and Yellow (structure; 10 replicate simulations, *K* = 1–19, 10^3^ burn-in, 10^6^ MCMC replicates, LOCPRIOR).

Population structure analysis using the Evanno method^28,29^ estimated the most likely number of genetic clusters (*K*) for *M*. *cavernosa* populations on the Belize Barrier Reef to be *K* = 3. *K* selection through log model likelihood (L(*K*)) was in agreement with the Evanno method (Supplementary Fig. S2). Based on these results, a structure bar plot was generated for *K* = 3 (highest (L(*K*)) and *∆K* value). In the *K* = 3 bar plot, the Blue or Green genetic cluster was the primary genetic cluster in all but one population (Raph’s Wall–35 m). Shallow populations (10 m and 16 m) primarily grouped to the Green genetic cluster (73.71–95.14% individual membership probabilities) while deep populations (25 m and 35 m) primarily grouped to the Blue genetic cluster (42.88–74.71%), with the exception of Raph’s Wall–35 m, which predominantly grouped to the Yellow genetic cluster (50.5%). While 25 m sample populations were genetically distinct from shallow populations, they had a stronger signal of admixture with shallow populations (Green cluster) than did 35 m populations (Fig. 5). Deep populations also had higher levels of admixture with the Yellow genetic cluster compared to shallow populations, and levels of admixture increased in prevalence from 25 m to 35 m sample populations.

### Vertical and horizontal migration models

At all sampling sites, vertical migration tests resulted in *Panmixia* models as the most likely scenario, yielding the highest model probabilities (Table 1). All other models (*Symmetric*, *Upward*, *Downward*) had probabilities near zero. Estimated mutation scaled population sizes (*Θ*) for all vertical migration tests at all four sites were an order of magnitude larger at Glover’s Reef and Raph’s Wall (15.30 and 21.43, respectively) compared to South Reef and Tobacco Reef (1.10 and 3.96, respectively). In horizontal migration tests a similar pattern was found, where *Panmixia* models resulted in model probabilities of one for both East/West (all sites) and North/South (South Reef, Raph’s Wall, Tobacco Reef) models with all other models (*Symmetric*, *Southward*, *Northward*, *Eastward*, *Westward*) having model probabilities near zero.

**Table 1:** Migration model rankings based on Bezier log marginal likelihood (lmL) comparisons.

Migration set	Model description	lmL	Model probability	Model rank	*Θ* (±95% CI)
TR vertical	*Symmetric*	−81177	0	4	
	*Downward*	−44217	0	3	
	*Upward*	−7151	0	2	
	*Panmixia*	0	1	1	3.96 (2.13–5.80)
RW vertical	*Symmetric*	−54509	0	4	
	*Downward*	−26709	0	3	
	*Upward*	−14807	0	2	
	*Panmixia*	0	1	1	21.43 (18.87–24.87)
SR vertical	*Symmetric*	−82409	0	4	
	*Downward*	−11180	0	2	
	*Upward*	−52940	0	3	
	*Panmixia*	0	1	1	1.10 (0–3.27)
GR vertical	*Symmetric*	−509986	0	2	
	*Downward*	−515363	0	3	
	*Upward*	−1362433	0	4	
	*Panmixia*	0	1	1	15.30 (12.80–18.33)
North/South	*Symmetric*	−54509.16	0	4	
horizontal	*Northward*	−26708.92	0	3	
	*Southward*	−14807.48	0	2	
	*Panmixia*	0	1	1	2.57 (0.53–4.60)
East/West	*Symmetric*	−81176.92	0	3	
horizontal	*Westward*	−44217.2	0	2	
	*Eastward*	−7151.01	0	4	
	*Panmixia*	0	1	1	11.9 (9.07–14.33)

Model probabilities are listed and ranked according to increasing likelihoods, with the most likely model for each migration set indicated by a ‘1’. Estimate of mutation-scaled population size (*Θ*) with 95% confidence intervals are listed for the most likely model. Vertical migration models were tested between “shallow” (10 m and 16 m) and “deep” (25 m and 35 m) populations at each sampling site denoted in Fig. 1. North/South horizontal connectivity models combined depth zones into a single population per site and horizontal migration was tested in South Water Caye Marine Reserve (SR, RW, TR; GR excluded). East/West horizontal connectivity models combined depth zones into a single population for sites in South Water Caye Marine Reserve (SR, RW, TR) and Glover’s Reef Marine Reserve (GR) and horizontal migration was tested between the marine reserves. Model descriptions as follows: *Symmetric*- symmetric migration; *Downward*- migration from shallow to deep; *Upward*- migration from deep to shallow; *Northward*- migration from south to north; *Southward*- migration from north to south; *Westward*- migration from east to west; *Eastward*- migration from west to east; *Panmixia*- a single panmictic population.

## Discussion

Populations of *M*. *cavernosa* in this study demonstrated a distinct shallow/deep genetic break, with strong genetic differentiation between a shallower population (10 m and 16 m) and adjacent deeper population (25 m and 35 m). This apparent genetic breaking point between 16 m and 25 m depth zones was consistent across all sites within South Water Caye Marine Reserve and also at Glover’s Reef Marine Reserve (~35 km east of South Water Caye Marine Reserve). All sampling sites had similar geomorphology, with “shallow” populations atop the reef (i.e. back reef and reef crest) and “deep” populations on the reef slope (fore reef and wall; Fig. 2). Additionally, the 35 m samples had higher probability of membership to a Yellow genetic cluster as compared to all other samples from other depth zones, potentially indicating a “depth-specialist” cluster with additional genetic structure beyond our sampling depths in the upper mesophotic depth zone. *M*. *cavernosa* have historically been identified deeper than 35 m in the region^20,26^, but additional samples beyond 35 m are required to assess any potential structure or specificity further into the mesophotic zone of Belize, as has been discovered in similar studies of other regions^15,30^.

While vertical connectivity was relatively low in the region, there was strong evidence for horizontal genetic connectivity within depth zones across the region. Results of all analyses indicated that *M*. *cavernosa* populations along the Belize Barrier Reef exhibit little horizontal structuring among sites. Pairwise *F*_ST_ comparisons revealed that Raph’s Wall populations were the most highly differentiated from populations at all other sampling sites (Fig. 4). This was unexpected given the similarity in geomorphology to other sites and the proximity of Raph’s Wall to South Reef and Tobacco Reef (0.796 km and 6.268 km, respectively) in comparison to the proximity of Glover’s Reef to all barrier reef sites (31.194–32.322 km; Fig. 1). Overall, it is evident that even though *M*. *cavernosa* populations in Belize are relatively well-mixed in terms of horizontal connectivity, deeper populations do not appear to be major larval contributors to shallow populations within the region, or vice versa. The similarity in population structure across these sampled sites may be driven by a number of regional abiotic and/or biotic factors, but more research is needed to elucidate the drivers of population differentiation across depth zones on the Belize Barrier Reef.

The coincidence of the genetic break with the shift in reef geomorphology may be indicative that the physical transition from reef crest to reef wall has an effect on the genetic structuring of *M*. *cavernosa* populations. Though it is unlikely that the geomorphology of the reef in this region is a direct physical barrier to gene flow, differences in geomorphology between depth zones likely influence abiotic and biotic factors which in turn contribute to the genetic structuring observed here. One such abiotic factor that may impede gene flow between *M*. *cavernosa* populations are small-scale oceanographic (i.e. <1 km) currents. There is previous evidence for spatial and temporal divergence in surface current regimes based on location on the Belize Barrier Reef. Hydrodynamic models suggest that eddy circulation direction affects cyclonic circulation around the Belize Barrier Reef subsequently influencing flow direction^31,32^. Models based on drifter releases have suggested that shoreward of the Belize Barrier Reef, currents typically demonstrate a westward motion, while seaward of the reef they show variability in motion (northeast to west) depending on the patterns of the predominant Yucatan current^33^. Additionally, surface current models from the region have demonstrated that small particles near Glover’s Reef Atoll tend to move northward while particles shoreward of South Water Caye tend to be retained on the Belize shelf ^34^. The discrepancy between shoreward and seaward currents is further accentuated during late summer as offshore current velocity increases^33^, which has potential implications for dispersal of larvae during spawning in late July through September. Given the available data on current dynamics and population structure of *M*. *cavernosa*, it is likely that deep gametes and larvae are transported northward, away from shallow gametes and larvae which are likely retained on the barrier reef^18^.

Temperature and light are among other abiotic factors that may influence *M*. *cavernosa* population structure on the Belize Barrier Reef. Temperature is an important abiotic factor for many marine species, especially scleractinian corals which are susceptible to bleaching events when water temperatures are elevated for prolonged periods^1,35,36^. Corals in MCEs have been found to have lower bleaching thresholds as compared to nearby shallow reefs^37^, though it is unclear whether this is a genotypic or phenotypic response. Light is also an important abiotic driver of ecological structure and function, especially in MCEs^6,10^. It has been suggested that rather than strict depth definitions of MCEs, light may be a better metric for defining MCEs, either directly or through the absence of light-dependent coral species^10,38^. Reef geomorphology can affect the optical properties between shallow and mesophotic habitat, with higher-relief reef sites having a significantly shallower 1% optical depth (i.e. the photosynthetic compensation point) than low-relief reef sites^10^. On the Belize Barrier Reef where reef slope may be near vertical or even inverted in some areas, the implications of geomorphology on optical characteristics result in shallower 1% and 10% (i.e. the midpoint of the euphotic zone) optical depths (58 and 28 m, respectively) as compared to other high-relief reef sites^10^. While some temperature and light data are available for Belize^39–41^, additional *in situ* data are needed from all depth zones, particularly into the upper mesophotic zone. This data coupled with *ex situ* experimentation would prove useful in evaluating these factors as potential drivers of genetic differentiation.

Asynchronicity in spawning between shallow and mesophotic *M*. *cavernosa* colonies may act as a prezygotic barrier, driving population differentiation and structure. As gonochoric broadcast spawners, male and female *M*. *cavernosa* synchronously release gametes that mix and fertilize in the water column or near the surface approximately one week after the full moon between the months of July and September^42,43^. Colonies at mesophotic depths have previously been observed to spawn simultaneously with shallow conspecifics, potentially allowing for inter-breeding between depth zones^44^. Only one study has examined spawning synchronicity of *M*. *cavernosa* across shallow and mesophotic depth gradients to date. At Flower Garden Banks National Marine Sanctuary in the NW GOM, shallow and mesophotic *M*. *cavernosa* colonies have been documented spawning in synchrony^44^. In Belize there is evidence for asynchronous spawning of broadcast spawning macrobenthic organisms between shallow and mesophotic depths, including the sponge *Xestospongia muta*^45^ and the scleractinian coral *Orbicella franksi*^25^. Mesophotic *X*. *muta* were observed spawning while no shallow conspecifics spawned^45^ and mesophotic *O*. *franksi* were found to spawn an average of 40 minutes prior to shallow conspecifics^25^. Asynchronicity in spawning can lead to reproductive isolation in coral populations, but temporal differences between conspecifics at different depths but may also allow deeper gametes time to reach the surface and mix with shallow gametes^46^. There is presently little information on spawning of *M*. *cavernosa* in MCEs in Belize, including any details of synchronicity with shallow conspecifics. There is also the possibility of interactive effects of spawning time and currents on vertical connectivity in this region. Gametes from mesophotic *M*. *cavernosa* may be susceptible to opposing currents on the seaward reef margin, transporting them away from local reefs while shallow gametes are being retained locally onto the barrier reef ^33,34^, leading to little chance of cross fertilization between shallow and deep conspecifics. This would result in a lack of gene flow between populations, even in the absence of other biological barriers between MCE and shallow conspecifics.

Genetic structure may also be affected through contemporary postzygotic barriers. It is possible that larvae of parent colonies from one depth zone may settle in depth zones different from their origin (e.g. deep larvae may settle on shallow reefs or vice versa). These larvae may be maladapted for environmental or biological factors at this alternate depth which may lead to decreased survivorship^47,48^, creating a lack of gene flow such as that which we have seen in *M*. *cavernosa* populations in Belize. In contrast, postzygotic genetic barriers could also occur pre-settlement. Coral larvae are known to use multiple cues to strategically settle on suitable reef habitat^49–51^. Coral larvae may be exhibiting microhabitat selection between sites on the reef crest or the reef slope to minimize phenotype–environment mismatches, thus increasing survivorship^47,52,53^. However, in a study on *O*. *franksi* Noren^25^ found that larvae from upper mesophotic colonies instead demonstrated preferential settlement on aragonite tiles conditioned at shallow depths. Although Noren^25^ found no evidence of postzygotic barriers to settlement in *O*. *franksi*, these data were recorded after only 4 weeks, which may be too soon to detect any effects of maladaptation of deep larvae to shallow habitats on the reefs in this region.

Although we were able to detect significant contemporary genetic differentiation between relatively deep and shallow *M*. cavernosa populations in Belize, migration analyses found little evidence for directional historical migration in any vertical or horizontal models, instead selecting panmixia as the most likely scenario in all instances. The generation time for *M*. *cavernosa* is unknown, but based on the size threshold for sexual maturity and the long lifespan of colonies^43,54^ predicted patterns of migration are likely estimated over the order of thousands of years^55^. These results suggest that barriers to migration across these depth zones may have developed more recently than migration analyses are able to predict. Again, this may be due to differences in oceanographic current regimes between depth zones as well as larval responses to environmentally or biologically driven selection. These analyses also highlight the fact that despite significant genetic differentiation across depth zones, as indicated by *F*_ST_ values, overall these levels of genetic differentiation were relatively low. Studivan and Voss found similar results when modeling *M*. *cavernosa* vertical migration between shallow and mesophotic habitats in the NW GOM^55^, Belize, and southwest Florida^18^. However, Serrano *et al*.^16^ found in their study of *M*. *cavernosa* populations across “shallow” (≤10 m), “intermediate” (15–20 m) and “deep” (≥25 m) habitat that models with shallow to deep migration were most probable, even in sites with apparently panmictic population structure.

This study provides evidence that it may be important to sample more frequently between depth zones in future studies, as in some regions population structure and genetic differentiation may not coincide precisely with the habitat labels researchers assign them, such as “mesophotic” or “shallow”. Here we found that even though populations were separated by ~10 m in both depth and horizontal distance, we could see strong evidence of genetic structuring. Although the *M*. *cavernosa* colonies we sampled at 25 m belong to “shallow reef” habitat based upon their depth zonation, from a genetic perspective they are distinct from other shallower (10 m and 16 m) colonies, which grouped together as we might expect given their depth zonation. At all sampling sites the 25 m populations had similar genetic structure to that of populations found in the upper mesophotic zone (35 m). This is potential evidence that habitat designations assigned to reef zones may be less dependent upon depth zone and should perhaps place more emphasis on differences in habitat characteristics and environmental factors which may vary across regions.

Our results indicate there is strong genetic differentiation between relatively shallow and deep *M*. *cavernosa* populations in Belize, therefore we suggest these populations should be managed to conserve and maintain coral populations across all depths. We observed little evidence of contemporary gene flow between deep and shallow populations on the Belize Barrier Reef, therefore it seems that in Belize neither local deep nor shallow populations are likely to serve as a viable refuge for the other. It is important to continue monitoring shallow communities while also incorporating deeper communities into assessments of ecosystem services. The need for monitoring of deeper populations may be especially important, since deep and shallow populations may not respond similarly to stress and disturbance. Although this study demonstrates impeded gene flow between relatively shallow and deep *M*. *cavernosa* populations in Belize, there is evidence of strong connectivity between mesophotic *M*. *cavernosa* populations in Belize and relatively shallow *M*. *cavernosa* populations nearly 1000 km away in the Dry Tortugas off the coast of Florida^18^. Therefore, Belize’s deeper reefs may be serving as refuges, but for distant shallow reefs rather than local shallow reefs. Considering these findings, international management efforts may be required to help maintain coral metapopulations across broad geographic scales. Overall, both “shallow” and “deep” *M*. *cavernosa* populations will likely continue to be important to long-term health and resilience of coral reefs through their contributions to genetic diversity in Belize and beyond.

## Methods

### Study site selection

This study focused on the Belize Barrier Reef surrounding Carrie Bow Cay, off the Belizean coast. The reefs in this area typically contain a steep slope which minimizes horizontal distance among sampling depth zones (Fig. 2). Similar to many locations in the TWA, *M*. *cavernosa* is ubiquitous along the Belize Barrier Reef, found from <1–95 m^20,27]^. *M*. *cavernosa* colonies were sampled from four sites for this study: three sites in South Water Caye Marine Reserve, near Carrie Bow Cay (South Reef, Raph’s Wall, and Tobacco Reef), and one site in Glover’s Reef Marine Reserve (Glover’s Reef), ~30 km southeast of Carrie Bow Cay (Fig. 2; Table 2). Sites were only selected for sampling if there was suitable reef habitat spanning all targeted depth zones and a sufficient abundance of *M*. *cavernosa* colonies to allow statistically robust sampling efforts while minimizing the likelihood of sampling clones.

**Table 2:** Site details for *Montastraea cavernosa* genotyped samples (*n*) near Carrie Bow Cay, Belize (*N* = 242), unique multi-locus genotypes (*n*_g_) used for analyses (total = 239), mean (±SE) observed heterozygosity (*H*_O_), and mean (±SE) expected heterozygosity (*H*_E_). GPS coordinates shown as degree decimal minutes (WGS84).

Site name	Latitude	Longitude	Depth zone	*n*	*n*_g_	*H*_O_	*H*_E_
Tobacco Reef (TR)	16° 49.946′N	88° 4.441′W	10 m	15	15	0.726 ± 0.055	0.712 ± 0.062
			16 m	15	15	0.650 ± 0.082	0.686 ± 0.059
			25 m	15	15	0.562 ± 0.073	0.683 ± 0.080
			35 m	15	15	0.624 ± 0.077	0.658 ± 0.086
Raph’s Wall (RW)	16° 46.564′N	88° 4.479′W	10 m	15	15	0.637 ± 0.060	0.684 ± 0.061
			16 m	15	15	0.593 ± 0.074	0.674 ± 0.065
			25 m	16	15	0.687 ± 0.079	0.716 ± 0.062
			35 m	15	15	0.577 ± 0.086	0.695 ± 0.065
South Reef (SR)	16° 46.137′N	88° 4.433′W	10 m	16	16	0.703 ± 0.065	0.723 ± 0.056
			16 m	15	14	0.630 ± 0.068	0.689 ± 0.061
			25 m	15	15	0.559 ± 0.081	0.669 ± 0.081
			35 m	15	15	0.659 ± 0.045	0.722 ± 0.060
Glover’s Reef (GR)	16° 45.323′N	87° 46.875′W	10 m	15	15	0.681 ± 0.077	0.715 ± 0.059
			16 m	15	15	0.562 ± 0.036	0.679 ± 0.059
			25 m	15	14	0.615 ± 0.052	0.697 ± 0.073
			35 m	15	15	0.631 ± 0.079	0.748 ± 0.051

### Coral sample collection

Approximately fifteen *M*. *cavernosa* colonies were sampled at each of four depth zones (back reef ~10 m; reef crest ~16 m; fore reef ~25 m; upper mesophotic ~35 m; Fig. 2) per reef site (*N* = 242). Sampled colonies were >1 m apart, which has been found to sufficiently reduce the likelihood of sampling clones from asexual fragmentation^16^. A small skeletal fragment containing coral tissue (~6 cm^2^) was removed from each colony by SCUBA divers with hammer and masonry chisel and subsequently placed into an individual, uniquely numbered zip-top bag. Colonies were photographed prior to sampling with scaled lasers (15 cm) for size reference. Upon returning to the surface, sample bags were placed into a cooler at ambient seawater temperature and transported back to the Smithsonian Carrie Bow Cay Field Station for processing within 3 hours. At Carrie Bow Cay each *M*. *cavernosa* fragment was photographed and subsamples were generated via chisel fragmentation. Excess coral skeleton, sponge tissue, crustose coralline algae, and macroalgae were removed to reduce the introduction of polymerase chain reaction (PCR) inhibitors and contaminating DNA into samples. *M*. *cavernosa* samples were preserved in TRIzol reagent and stored at −20 °C until transportation back to FAU Harbor Branch on ice. Once in the laboratory, samples were stored at −80 °C until DNA extraction. Samples were collected over two field expeditions; samples from 35 m depth zones within South Water Caye Marine Reserve at (i.e. Tobacco Reef, Raph’s Wall, and South Reef) were collected in March 2016 by Studivan and Voss^18^, and samples from the remaining depth zones in South Water Caye Marine Reserve were collected in March 2017 (including resampling of 16 m depth zones). Samples from all depth zones in Glover’s Reef Marine Reserve were collected in March 2017.

### Genomic DNA extraction

Genomic DNA was extracted from the 2016 samples using a modified TRIzol extraction^56^ as described in Studivan and Voss^18^. For all 2017 samples a cetyl trimethylammonium bromide (CTAB) extraction was implemented following Mieog *et al*.^57^ with the following modifications: coral fragments were removed from thawed TRIzol and tissue (~0.1 g) was scraped from several polyps with a sterile scalpel. Tissue was placed into a 2.0 mL microcentrifuge tube with 800 µL of 2% CTAB extraction buffer, ~0.075 g of 0.5 mm glass beads, and 20 µg mL^−1^ proteinase K. The sample was macerated in a FastPrep®−24 homogenizer (MP Biomedicals) at 6 m s^−1^ for three 45 s intervals with a 2 min cool down period between intervals. The sample was incubated in a thermomixer at 60 °C while mixing (1000 rpm for 5 s, 1.5 min intervals) for 90 min. Following incubation, DNA extraction was performed according to the procedures outlined in Mieog *et al*.^57^, and samples were eluted in 100 µL 55 °C 1X TE (pH 8.0) and incubated for 10 min at 55 °C. Extracted DNA was cleaned using the Zymo Research DNA Clean & Concentrator™−5 kit to remove polysaccharides and other PCR inhibitors. Concentration and quality of cleaned DNA were measured on a NanoDrop™ 2000 (Thermo Fisher Scientific) spectrophotometer and samples were subsequently diluted for PCR amplification.

### *Montastraea cavernosa* microsatellite genotyping

Nine previously developed microsatellite loci^16^ were amplified to genotype *M*. *cavernosa* samples. Triplexed PCRs were used with self-labeled fluorescent primers (6FAM, VIC, and NED), which anneal to universal tails included in forward primers^55,58,59^, (Supplementary Table S1) using the QIAGEN Type-It Microsatellite PCR kit. PCRs were run following the methods outlined in Studivan and Voss^55^, except with 30 cycles per reaction. Amplified samples were visualized on a 2% agarose gel and diluted to 1:30–1:50 in deionized water based on band intensity from agarose gel images, prior to sequencing on an Applied Biosystems ABI 3130xl genetic analyzer with 500 ROX size standard. Alleles were scored from returned electropherograms using GeneMapper v3.7 (Applied Biosystems). Any electropherograms with ambiguous or non-existent allele peaks, or low quality size standard scores were re-amplified and re-screened (including re-extraction of DNA as necessary) to ensure consistent allele scoring, resulting in the most complete dataset possible. Samples were run an average of 1.1 times on the genetic analyzer.

### Assumption testing and population structure analyses

After alleles were scored, GenAlEx v6.503^60^ was used to identify 3 clonal multi-locus genotypes (MLGs) within sites, which were removed from all subsequent analyses (unique MLGs per population listed as *n*_g_ in Table 2). MLGs were determined to be the result of asexual reproduction rather than resampling of the same colony using reference photographs of sampled colonies or random chromosome recombination (all *p*-values < 0.0001) using MLGsim v2.0^61,62^ (1000 simulations). GenAlEx was used to calculate allele frequencies, evaluate populations for Hardy-Weinberg equilibrium for all loci, and calculate fixation indices (*F*_ST_). Linkage disequilibrium was calculated for all pairwise comparisons of loci among populations using Arlequin v3.5^63^. Hardy-Weinberg equilibrium and linkage disequilibrium *p-*values were corrected for false discovery rate (FDR) with the *R* package *fdrtool*^64^. No populations or loci were found to have significant patterns in violations of Hardy-Weinberg equilibrium (Supplementary Table S2) or linkage disequilibrium among loci within any populations. FreeNA^65^ was used to test the effects of null alleles on deviations from Hardy-Weinberg equilibrium. Null allele corrected and raw *F*_ST_ values by locus and population were tightly correlated (*R*^2^ = 0.996 and *R*^2^ = 0.985, respectively) therefore uncorrected *F*_ST_ values were used in subsequent analyses. Population differentiation was assessed with an analysis of molecular variance (AMOVA) in GenAlEx using fixation indices (*F*_ST_; 9999 model and population permutations). Pairwise *F*_ST_ estimates and *p*-values were first calculated for all sites and depths. Based on patterns from initial analysis of all sites and depths as discrete populations, additional analyses were conducted combining the relatively shallow sampling depths (10 m and 16 m) and the relatively deep sampling depths (25 m and 35 m) into “shallow” and “deep” populations, respectively, increasing population sample sizes and simplifying pairwise comparisons. Population differentiation was visualized with principal coordinates analysis (PCoA) using Nei genetic distance (D_A_) in GenAlEx. Isolation by geographic distance was assessed between all sample populations with a Mantel test (9999 permutations) in GenAlEx, using general site GPS coordinates to calculate linear distance between sites.

Population structure (i.e. genetic clusters; *K*) was estimated using Bayesian model-based clustering with the program structure v2.3.4^66^. The *R* package *ParallelStructure*^67^ was used to run structure in parallel on FAU’s high performance computing cluster, testing 10 replicate simulations for all values of *K* between 1–19 (maximum *K* = sampling sites + 3) to identify any possible cryptic genetic clusters within populations. All simulations used 10^3^ burn-in iterations and 10^6^ Markov Chain-Monte Carlo replicates, including the LOCPRIOR option which aids simulation testing based on sampling location. The web-based version of structure harvester^29^ was used to determine the most likely value of *K* through implementation of the Evanno method of *K* selection^28^, including the *ad hoc* test statistic *∆K*. A mean model simulation was generated for the most likely value of *K* as determined by structure harvester using clumpp v1.1.2^68^ based on replicate simulation runs, and population structure bar plots showing individual percent membership to genetic clusters were generated using distruct v1.1^69^.

### Vertical and horizontal migration modeling

Historical (~4 *N*_e_ generations) migration rates across vertical and horizontal spatial scales were estimated using migrate v3.6, which uses a Bayesian approach to estimate the mutation rate and determine the likelihood of population gene flow models through coalescence theory^70,71^. *A priori* migration models were developed to test (1) vertical migration within individual sites, (2) North/South horizontal migration along the barrier reef sites (South Reef, Raph’s Wall, Tobacco Reef), and (3) East/West horizontal migration between Glover’s Reef Atoll and the Belize Barrier Reef. When testing vertical migration, populations were combined into “shallow” (10 m and 16 m) and “deep” (25 m and 35 m) populations based on PCoA and *F*_ST_ results. The following set of migration models were used to determine (1) vertical migration at each sampling site: (A) *Symmetric*; full model with symmetric migration between depths, (B) *Upward*; asymmetric migration from deep to shallow depths, (C) *Downward*; asymmetric migration from shallow to deep depths, (D) *Panmixia*. Tests of (2) North/South horizontal migration used the following set of models: (A) *Symmetric*; full model with symmetric migration between sites, (B) *Southward*; asymmetric migration from northern to southern sites, (C) *Northward*; asymmetric migration from southern to northern sites, (D) *Panmixia*. Tests of (3) East/West horizontal migration used the following set of models: (A) *Symmetric*; full model with symmetric migration between sites, (B) *Westward*; asymmetric migration from eastern (Glover’s Reef) to western sites (South Reef, Raph’s Wall, Tobacco Reef), (C) *Eastward*; asymmetric migration from western to eastern sites, (D) *Panmixia*.

All migration simulations were run with the following parameters: long-inc 100, long-sample 15000, 20 replicates, burn-in 20000, and four heated chains of 1, 1.5, 3, 10^5^. Prior distributions for *Θ* (mutation-scaled population size) and *M* (mutation-scaled immigration rate) were set from 0–100 and 0–1000, respectively. For each set of migration models, the best model simulation was chosen based upon comparison and ranking of Bezier log marginal likelihoods using the thermodynamic integration method detailed by Beerli and Palczewski^70^.

## Supplementary information

Supplementary Information

Supplementary Dataset

## Data Availability

Microsatellite genotype data and structure output files are available as a Supplementary Dataset file. Additional analysis scripts and documentation are available on GitHub (https://github.com/mstudiva/Mcav-microsats.git).
