# Supplementary material for: Populations of the coral species Montastraea cavernosa on the Belize Barrier Reef lack vertical connectivity
Source: Sci Rep. 2019 May 10;9:7200. doi: 10.1038/s41598-019-43479-x (PMC6510931; doi:10.1038/s41598-019-43479-x)
Supplement: Supplementary file 1 — Supplementary Information [file 41598_2019_43479_MOESM1_ESM.pdf]

Populations of the coral species *Montastraea cavernosa* on the Belize Barrier Reef lack vertical connectivity

Ryan J. Eckert, Michael S. Studivan, and Joshua D. Voss

Supplementary Information

Supplementary Table S1. *Montastraea cavernosa* triplex microsatellite loci primer sequences[1,2]. Allele size ranges listed include universal tail lengths[3].

Locus	Motif	Primer sequence (5'–3')	Tail	Tail sequence (5'–3')	Allele size w/ tail (bp)	Locus missing rate	Plex
MC29	(AAAC)7	F:CAGGACCAGGCTACCGTGCTCCTTGGTCACCCTACAA	C	NED-CAGGACCAGGCTACCGTG	173–212	0.00%	1
		R:GGTGAAGAAGCAGCCATTGG					
MC41	(GGTA) imperfect	F:GCCTCCCTCGCGCCAAATTACGCAACACTGTGCA	A	6FAM-GCCTCCCTCGCGCCA	359–463	0.84%	1
		R:TCGACTGACCGAAGTACCT					
MC49	(TGT)10	F:GCCTTGCCAGCCCGCATTCCTCCAGTGATGTACCT	B	VIC-GCCTTGCCAGCCCGC	207–399	1.67%	1
		R:CTGAGTTCCTGCCATTAGG					
MC46	(TTTTGT) imperfect	F:CAGGACCAGGCTACCGTGCGGTGTAGCTCTAGCAGGA	C	NED-CAGGACCAGGCTACCGTG	142–181	0.00%	2
		R:ACTGAGTCGCAGCATTTGG					
MC65	(TTTGGT)6	F:GCCTCCCTCGCGCCATTTGTGATTGGCCAGGGTG	A	6FAM-GCCTCCCTCGCGCCA	127–187	0.00%	2
		R:TTGTGCTGTGAAGCATGAT					
MC97	(ACAA)6 ACAG (ACAA)	F:GCCTTGCCAGCCCGCACATGTGGCCTTGTTACCA	B	VIC-GCCTTGCCAGCCCGC	178–202	1.26%	2
		R:CGAACATCAGTGACAACCT					
MC4	(TTA)7 T (TTA)2	F:CAGGACCAGGCTACCGTGACGATCAAGACTCCAACGA	C	NED-CAGGACCAGGCTACCGTG	115–248	5.02%	3
		R:GCTCTTCGTGAACACTGAGG					
MC18	(AAT)2 TAT (AAT)9	F:GCCTCCCTCGCGCCAGGAGAACTGGATACCATGTC	A	6FAM-GCCTCCCTCGCGCCA	233–275	4.18%	3
		R:TATGGTCCTGGGACAACTT					
MC114	(TTG)10 [15bp insert] (TTG)6	F: GCCTTGCCAGCCCGCACTGTAGATCGAGGCGTTTC	B	VIC-GCCTTGCCAGCCCGC	167–245	0.84 %	3
		R: TCTGTTCCTCTGACTCTTTCG					

Supplementary Table S2. Summary of genetic diversity statistics across loci by population. n_g, number of unique multi-locus genotypes with amplified alleles; n_a, number of alleles; H_O, observed heterozygosity; H_E, expected heterozygosity; *p*HWE, *FDR*-corrected *p*-value for tests of Hardy-Weinberg equilibrium (HWE; insignificant tests shown as ns).

Site	Depth (m)	Statistic	MC4	MC18	MC29	MC41	MC46	MC49	MC65	MC97	MC114
Tobacco Reef (TR)	10	n_g	15	15	15	15	15	15	15	15	15
		n_a	11	9	6	7	4	7	4	5	11
		H_O	0.733	0.867	0.8	0.867	0.533	0.8	0.4	0.667	0.867
		H_E	0.889	0.86	0.778	0.731	0.429	0.747	0.391	0.696	0.889
		*p*HWE	ns	ns	ns	ns	ns	ns	ns	ns	ns
	16	n_g	12	15	15	15	15	15	15	15	15
		n_a	14	10	5	4	4	8	4	5	11
		H_O	0.917	0.867	0.6	0.667	0.267	0.6	0.267	0.867	0.8
		H_E	0.91	0.853	0.693	0.713	0.34	0.671	0.509	0.647	0.833
		*p*HWE	ns	ns	ns	ns	ns	ns	ns	ns	ns
	25	n_g	14	15	15	15	15	15	15	15	15
		n_a	21	8	6	6	2	8	3	5	11
		H_O	0.929	0.333	0.533	0.6	0.333	0.667	0.267	0.733	0.667
		H_E	0.941	0.836	0.72	0.684	0.358	0.838	0.238	0.651	0.88
		*p*HWE	ns	**<0.001**	ns	ns	ns	ns	ns	ns	ns
	35	n_g	13	15	15	14	15	14	15	13	15
		n_a	20	7	7	5	3	8	2	5	11
		H_O	0.923	0.733	0.8	0.571	0.467	0.714	0.133	0.538	0.733
		H_E	0.944	0.778	0.733	0.651	0.371	0.809	0.124	0.642	0.871
		*p*HWE	ns	ns	ns	ns	ns	ns	ns	ns	ns
Raph's Wall (RW)	10	n_g	15	15	15	15	15	15	15	15	15
		n_a	13	8	7	6	4	5	2	6	9
		H_O	0.8	0.733	0.8	0.667	0.4	0.467	0.4	0.6	0.867
		H_E	0.882	0.811	0.722	0.784	0.42	0.48	0.444	0.762	0.853
		*p*HWE	ns	ns	ns	ns	ns	ns	ns	ns	ns
	16	n_g	15	15	15	15	15	15	15	15	15
		n_a	15	8	6	5	5	6	4	5	10
		H_O	0.733	0.6	0.4	0.533	0.533	0.467	0.267	0.8	1
		H_E	0.924	0.811	0.676	0.72	0.484	0.589	0.296	0.707	0.856
		*p*HWE	ns	ns	ns	ns	ns	ns	ns	ns	ns

Site	Depth (m)	Statistic	MC4	MC18	MC29	MC41	MC46	MC49	MC65	MC97	MC114
Raph's Wall (RW)	25	n_g	15	14	15	15	15	15	15	15	15
		n_a	19	7	8	5	3	9	4	6	11
		H_O	0.933	0.714	0.933	0.6	0.467	0.533	0.267	0.8	0.933
		H_E	0.936	0.783	0.778	0.682	0.451	0.816	0.389	0.718	0.893
		*p*HWE	ns	ns	ns	ns	ns	ns	ns	ns	ns
	35	n_g	15	15	15	15	15	15	15	14	15
		n_a	19	8	6	5	3	8	4	5	10
		H_O	0.8	0.4	0.8	0.4	0.267	0.267	0.533	0.929	0.8
		H_E	0.936	0.813	0.787	0.509	0.331	0.751	0.527	0.742	0.856
		*p*HWE	ns	ns	ns	ns	ns	**<0.001**	ns	ns	ns
South Reef (SR)	10	n_g	14	15	16	16	16	16	16	16	15
		n_a	15	10	9	6	4	7	4	5	12
		H_O	0.857	0.933	0.75	0.75	0.375	0.5	0.625	0.938	0.6
		H_E	0.911	0.871	0.725	0.758	0.412	0.545	0.633	0.75	0.9
		*p*HWE	ns	ns	ns	**0.002**	ns	**0.001**	ns	ns	ns
	16	n_g	11	13	14	14	14	14	14	14	14
		n_a	13	8	5	5	3	6	3	6	8
		H_O	0.545	0.692	0.571	0.571	0.5	0.643	0.286	0.929	0.929
		H_E	0.897	0.822	0.61	0.747	0.439	0.73	0.36	0.763	0.832
		*p*HWE	ns	ns	ns	ns	ns	ns	ns	ns	ns
	25	n_g	15	13	15	15	15	15	15	15	14
		n_a	19	7	7	7	2	7	4	5	11
		H_O	0.733	0.308	0.733	0.467	0.133	0.667	0.4	0.733	0.857
		H_E	0.922	0.793	0.744	0.6	0.124	0.787	0.473	0.736	0.839
		*p*HWE	ns	ns	ns	ns	ns	ns	ns	ns	ns
	35	n_g	15	13	15	14	15	13	15	15	15
		n_a	21	7	8	8	2	10	4	5	10
		H_O	0.8	0.615	0.8	0.5	0.533	0.615	0.533	0.667	0.867
		H_E	0.942	0.784	0.784	0.742	0.444	0.858	0.42	0.671	0.849
		*p*HWE	ns	ns	ns	**0.001**	ns	ns	ns	ns	ns
Glover's Reef (GR)	10	n_g	15	15	15	15	15	15	15	15	15
		n_a	16	10	6	6	2	9	5	6	13
		H_O	0.933	0.733	0.667	0.667	0.2	0.533	0.667	0.733	1
		H_E	0.911	0.869	0.691	0.724	0.358	0.776	0.616	0.598	0.891
		*p*HWE	ns	ns	ns	ns	ns	ns	ns	ns	ns

Site	Depth (m)	Statistic	MC4	MC18	MC29	MC41	MC46	MC49	MC65	MC97	MC114
Glover's Reef (GR)	16	n_g	14	15	15	15	15	14	15	15	15
		n_a	13	9	5	4	3	6	5	5	9
		H_O	0.5	0.533	0.733	0.6	0.467	0.429	0.533	0.533	0.733
		H_E	0.893	0.82	0.718	0.738	0.371	0.676	0.518	0.522	0.858
		*p*HWE	ns	ns	ns	ns	ns	ns	ns	ns	ns
	25	n_g	14	13	14	14	14	14	14	14	14
		n_a	18	8	7	6	3	8	4	4	11
		H_O	0.786	0.538	0.643	0.643	0.429	0.571	0.357	0.786	0.786
		H_E	0.934	0.802	0.773	0.755	0.357	0.801	0.311	0.666	0.872
		*p*HWE	ns	ns	ns	ns	ns	ns	ns	ns	ns
	35	n_g	15	14	15	15	15	15	15	15	15
		n_a	19	7	7	6	4	7	4	7	9
		H_O	0.733	0.143	0.733	0.6	0.533	0.867	0.467	0.667	0.933
		H_E	0.931	0.816	0.836	0.707	0.453	0.82	0.553	0.778	0.84
		*p*HWE	ns	**<0.001**	ns	ns	ns	ns	ns	ns	ns

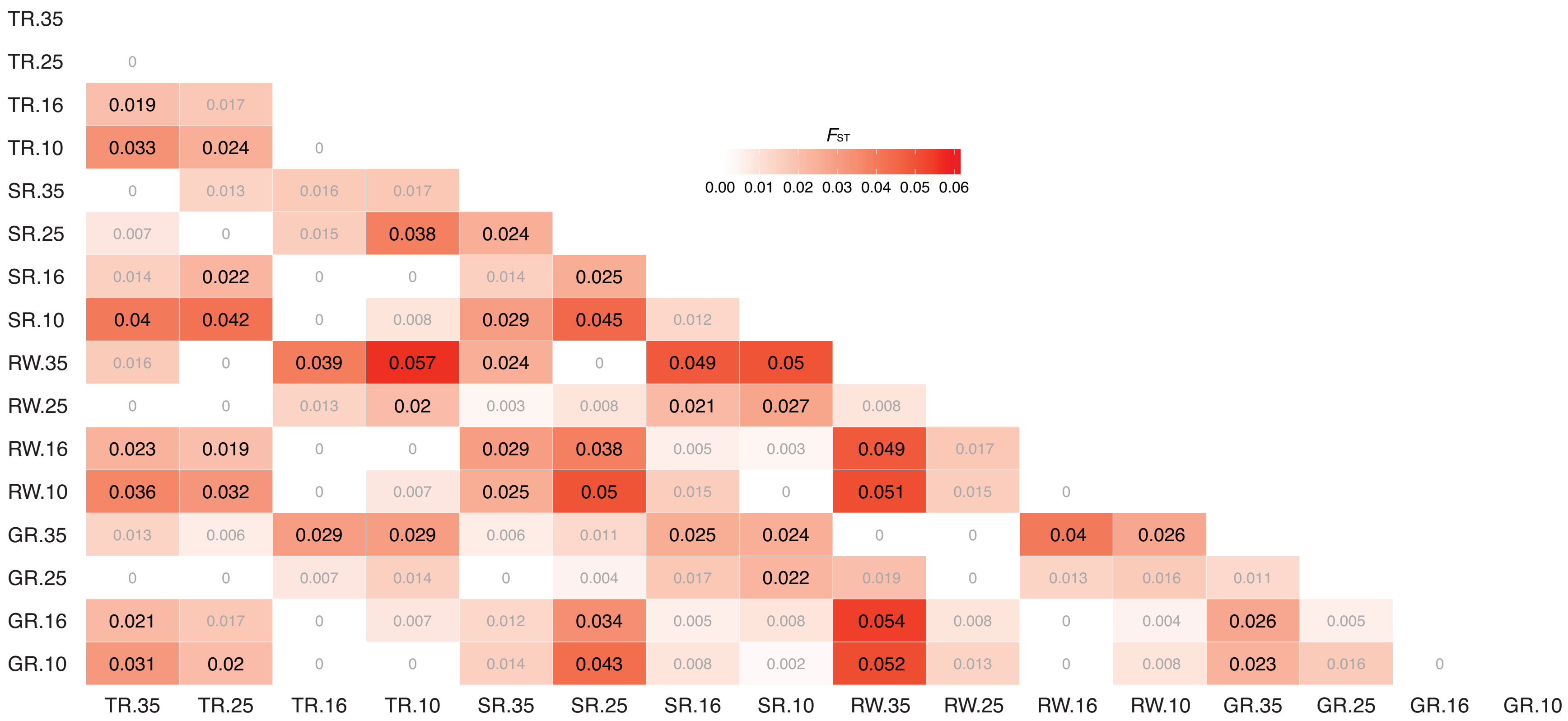

Supplementary Figure S1. Heat map showing pairwise sample population differentiation through estimation of fixation index (F_{ST}). Values within cells are estimated F_{ST}; intensity of red coloration corresponds to increasing F_{ST}; bolded values denote significant differentiation between populations (after FDR correction; $\alpha = 0.05$). Site abbreviations listed in Table 1.

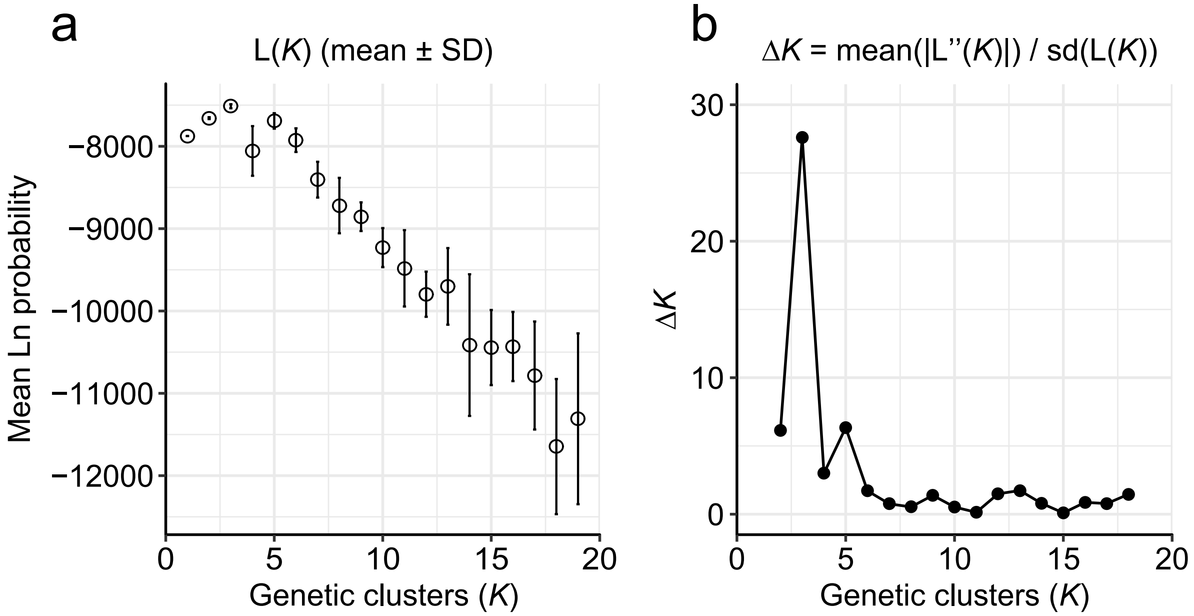

Supplementary Figure S2. Plots showing (a) mean Ln probability and (b) Δ*K* values, used in selection of most likely number of genetic clusters (*K*) for values of *K* = 1–19. Error bars represent SD of the mean.

References

1. Serrano, X. *et al.* Geographic differences in vertical connectivity in the Caribbean coral *Montastraea cavernosa* despite high levels of horizontal connectivity at shallow depths. *Mol. Ecol.* **23**, 4226–4240, doi: 10.1111/mec.12861 (2014).
2. Studivan, M. S. & Voss, J. D. Assessment of Mesophotic Coral Ecosystem Connectivity for Proposed Expansion of a Marine Sanctuary in the Northwest Gulf of Mexico: Population Genetics. *Front. Mar. Sci.* **5**, 1–11, doi: 10.3389/fmars.2018.00152 (2018).
3. Blacket, M. J., Robin, C., Good, R. T., Lee, S. F. & Miller, A. D. Universal primers for fluorescent labelling of PCR fragments-an efficient and cost-effective approach to genotyping by fluorescence. *Mol. Ecol. Resour.* **12**, 456–463, doi: 10.1111/j.1755-0998.2011.03104.x (2012).
